# A full-STAC remedy for global digital health transformation: open standards, technologies, architectures and content

**DOI:** 10.1093/oodh/oqad018

**Published:** 2023-12-15

**Authors:** Garrett L Mehl, Martin G Seneviratne, Matt L Berg, Suhel Bidani, Rebecca L Distler, Marelize Gorgens, Karin E Kallander, Alain B Labrique, Mark S Landry, Carl Leitner, Peter B Lubell-Doughtie, Alvin B Marcelo, Yossi Matias, Jennifer Nelson, Von Nguyen, Jean Philbert Nsengimana, Maeghan Orton, Daniel R Otzoy Garcia, Daniel R Oyaole, Natschja Ratanaprayul, Susann Roth, Merrick P Schaefer, Dykki Settle, Jing Tang, Barakissa Tien-Wahser, Steven Wanyee, Fred Hersch

**Affiliations:** Digital Health and Innovation, World Health Organization, 20 Avenue Appia, 1211 Geneva, Switzerland; Google Health, Google Inc, Belgrave House, 76 Buckingham Palace Road, London SW1W, UK; Ona, 2nd floor, George Padmore Ln, Nairobi, Kenya; India Country Office, Bill and Melinda Gates Foundation, Capital Court, The, 5th Floor, Olof Palme Marg, Munirka, New Delhi, Delhi 110067, India; The Patrick J. McGovern Foundation, PO Box 171536 Boston, MA 02117-3375, USA; Health, Nutrition and Population Global Practice, World Bank Group, 1818 H Street NW, Washington, DC 20433, USA; Health Programme Group, UNICEF, 3 UN Plaza, New York, NY 10017, USA; Digital Health and Innovation, World Health Organization, 20 Avenue Appia, 1211 Geneva, Switzerland; MECA, The Global Fund to Fight AIDS, Tuberculosis and Malaria, Chem. du Pommier 40, 1218 Le Grand-Saconnex, Switzerland; Digital Health and Innovation, World Health Organization, 20 Avenue Appia, 1211 Geneva, Switzerland; Ona, 2nd floor, George Padmore Ln, Nairobi, Kenya; Medical Informatics Unit, University of the Philippines, 547 Pedro Gil Street, Ermita Manila, Philippines; Google Research, Google Inc, 98 Alon Yigal TEL AVIV-JAFFA, 6789141, Israel; Social Protection and Health, Inter-American Development Bank, USA; Google Health, Google Inc, 1105 West Peachtree St View, Atlanta, Georgia, USA; Africa Centres for Disease Control and Prevention African Union Commission, Roosevelt Street W21 K19, P.O. Box 3243, Addis Ababa, Ethiopia; Global Health Academy, Old Medical School (Doorway 3), Teviot QuadTeviot Place, University of Edinburgh, Edinburgh EH8 9AG, Scotland, UK; RECAINSA, RECAINSA, 23 calle 14-58 zona 4 de Mixco Penthouse Edificio Crece Torre 1 Oficina 1104, Guatemala City, Guatemala; Digital Health, GAVI Vaccine Alliance, Chem. du Pommier 40, 1218 Le Grand-Saconnex, Switzerland; Digital Health and Innovation, World Health Organization, 20 Avenue Appia, 1211 Geneva, Switzerland; Asian Development Bank, 6 ADB Avenue, Mandaluyong City 1550, Metro Manila, Philippines; Digital Health, United States Agency for International Development, 1300 Pennsylvania Ave, NW. Washington DC 20004, USA; PATH, 2201 Westlake Ave Suite 200, Seattle, WA 98121, USA; Google Health, Google Inc, Belgrave House, 76 Buckingham Palace Road, London SW1W, UK; Sector Initiative Global Health, Deutsche Gesellschaft für Internationale Zusammenarbeit (GIZ) GmbH, Bonn, Germany; Health Informatics in Africa (HELINA), Department of Biostatistics, School of Public Health, University of Ghana, Legon, Accra P.O Box LG 13 Legon Accra, GA/R, Ghana; Google Health, Google Inc, 70 Pasir Panjang Rd, #03-71 Mapletree Business City II, 117371, Singapore

**Keywords:** health informatics, interoperability standards, health systems, global strategy on digital health, HL7 FHIR, digital transformation

## Abstract

The global digital health ecosystem is project-centric: point solutions are developed for vertical health programs and financed through vertical funding allocations. This results in data fragmentation and technology lock-in, compromising health care delivery. A convergence of trends enabled by interoperability and digital governance makes possible a shift towards person-focused health. Together, open Standards, open Technologies, open Architectures and open Content represent a next-generation ‘full-STAC’ remedy for digital health transformation. Local developers and implementers can avoid reinventing the wheel, and instead build digital tools suited to local needs—where data travels with an individual over time, evidence-based practice is easily integrated, and insights are gleaned from harmonized data. This is the culmination of the vision endorsed by 194 WHO Member States in the Global Strategy on Digital Health 2020 to 2025.

Digital technologies are quickly becoming the backbone for delivery of health care around the world. In low-resource and emergency settings that account for some of the hardest-to-reach communities, they are an essential tool for delivering care at scale and play a key role in efforts to address important health equity gaps [26]. We have seen successful digital health implementations across diverse geographies and use-cases, from non-communicable disease screening [9] to antenatal care [7], humanitarian response [29] and outbreak surveillance [24]. During the COVID-19 pandemic, mobile vaccination certificates and electronic immunization registries reinforced the potential of digital tools in low-resource settings [23]. Despite this, the global digital health sector has been dominated by point solutions developed for vertical health programs and financed through vertical funding allocations devoid of interoperability standards, resulting in a number of systemic challenges that have held back the field. These include fragmented data, redundant investments, delays in the dissemination of evidence-based practice, the scourge of digital health ‘pilotitis’ [18], accumulation of technical debt [48] and technology lock-in [17]. A 2020 USAID policy document decried such ‘investments in individual, disease-focused and non-interoperable tools’ and proposed a pathway to investing differently [46]. Without a radical rethinking in the way technologies are designed, governed and assessed and investments are prioritized, and recognition for the centrality of interoperability standards, the promise of digital health transformation—to meaningfully address health equity gaps and improve health outcomes at scale—cannot be realized. A rationalized approach to system architecture—one that leverages open semantic and syntactic standards—enables the possibility for a sustainable, linear growth in costs of digital transformation as a function of the number of systems connected, rather than the current state of unsustainable, quadratic growth in costs.

Today, an unprecedented convergence of emerging solutions remedies prior shortfalls and sets the stage for interoperable, financially and technically sustainable digital investments—bringing us closer to a reality of evidence-based, implementable, person-focused and digitally enabled health for all. In this vision, an ecosystem of locally developed or adapted, maintained and supported digital health tools plug into a standards-based longitudinal health record that supports the patient over time [21] and across places where interactions with the health system occurs, collects shareable data and responds to changing treatment guidelines. This aligns with goals articulated in international policy frameworks and action plans, including the Global Strategy on Digital Health endorsed by the 194 WHO Member States at the World Health Assembly in 2020 [48], the UN Roadmap for Digital Cooperation [45] and the Principles for Digital Development [38]), which collectively outline the digital enablers to achieving the Sustainable Development Goals and Universal Health Coverage [44]. This vision is now within reach due to a convergence of four factors—open Standards, open Technologies, open Architectures and open Content—which together represent a next-generation ‘full-STAC’ approach for global digital health transformation (Fig. 1). The open nature of these four factors is essential in creating the enabling environment for a large, transparent and competitive marketplace of digital health vendors, which can act locally, regionally, or globally as engines of digital health transformation [8].

**Figure 1:**
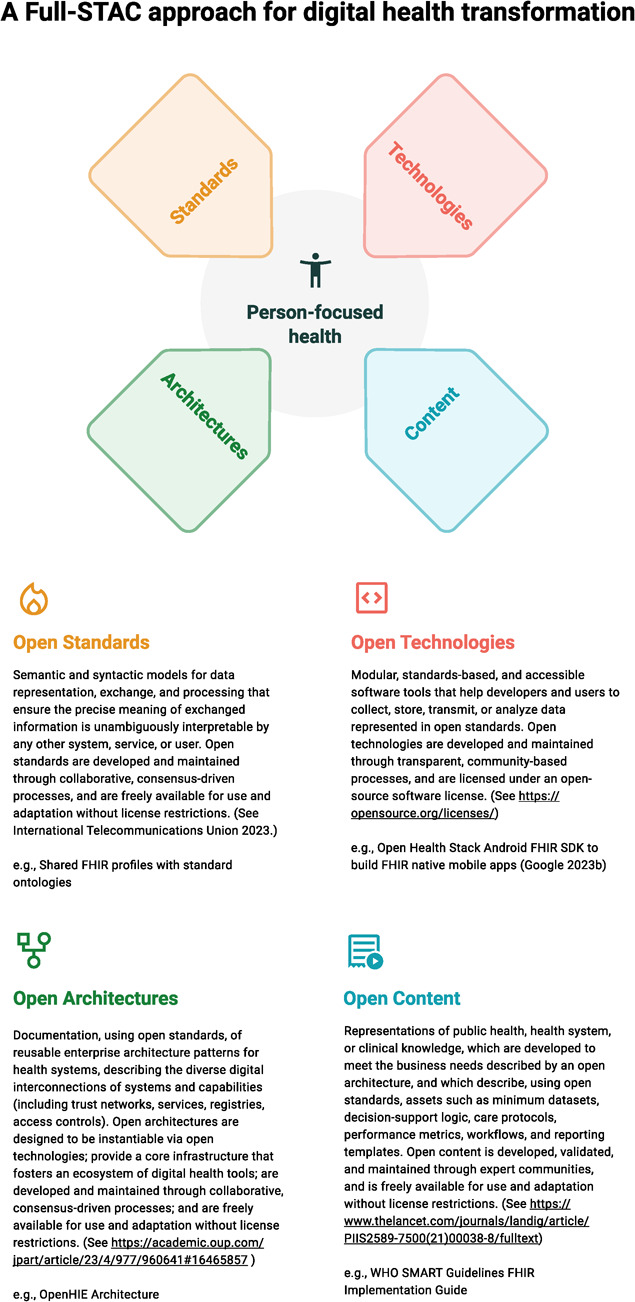
A ‘full-STAC’ remedy for global digital health transformation comprising open standards, technologies, architectures and content [8, 11, 20, 28, 36].

OPEN STANDARDS refers to a common schema for health data, calculations and information exchange. The Health Level Seven Fast Healthcare Interoperability Resource (HL7 FHIR®) standard is being widely adopted because it is open-access, well-documented and based on well-established technologies (Health Level Seven International 2023) [15]. These include RESTful application programming interfaces, XML and JSON, which make it easier for developers not traditionally working in the health-IT sector to implement and use [35]. HL7 FHIR profiles and implementation guides add an extra layer of interoperability by marrying the HL7 FHIR format with free- and open-standard terminologies such as the International Classification of Diseases (ICD) [52], Logical Observation Identifiers Names and Codes (LOINC) [39] or Systematized Nomenclature of Medicine Global Patient Set (SNOMED GPS) [42]. FHIR profiles accompanied by multiple semantic standards are now being incorporated into global [49] and regional guidance [1], and national e-health policies in the United States [34], India [30], Indonesia [31], Argentina [2, 22] and many other countries, and recommended by development partners [49, 47]. FHIR also works well with a second emerging standard; HL7 Clinical Quality Language (CQL), a human-readable query language to encode calculations for computing indicators and clinical logic to drive decision support [12]. Modality-specific standards, such as DICOM for imaging studies, can complement this FHIR-based framework [27]. Together, these standards make it possible to represent data and decision-making logic in a consistent, open and modular fashion [14].

The reality of FHIR, as with any new standard, is that it can be challenging to adopt for software developers. OPEN TECHNOLOGIES address the common technical and financial burdens software developers face, providing open-access software infrastructure that make it easier to build solutions that leverage the FHIR and CQL standards. These include servers to store, query and transform FHIR data [41]; form-building tools for authoring questionnaires [33]; tools to generate synthetic data [43]; and many others (Health Level Seven International 2021a) [13]. A notable example is Open Health Stack (OHS) [10], built in collaboration with a growing community of developers [5]. OHS consists of a new suite of digital public goods [6], including a software development kit for building FHIR-native apps on Android, and analytics tooling to generate insights from FHIR data [16]. This coding infrastructure is tailored to resource-constrained settings, with offline functionality and support for on-premise deployment. Such open technologies reduce the barrier to entry for new developers, enabling innovators everywhere to build or easily adapt FHIR-based solutions to solve local problems. Open technologies are open-source and use open standards (International Telecommunications Union, 2023). In thinking about open technologies, the certification and accreditation of solutions that use these technologies and adhere to open data standards in countries is essential [51].

For these FHIR-based technologies to enable a longitudinal person-centric approach, we also need open architectures. OPEN ARCHITECTURES specify the digital interconnections of a complex health system, including the trust networks, services and registries, and access controls that have been shown to improve quality and cost-effectiveness in health systems [40]. An example of this is the Open Health Information Exchange (OpenHIE), a community-driven architecture specifying the web of foundational components (e.g. health worker registry, logistics management service or shared health record), external point-of-service applications (via identity management, authentication, registry queries, etc.) and how they all fit together with one another [37]. These reusable architectural patterns are the ‘connective tissue’ required for countries to achieve nationally scaled, interoperable digital health solutions[Fn fn1]. Another example is the India Ayushman Bharat Digital Health Mission [30], a country-led effort to create an open architecture that includes certification and accreditation specifications for assessing software conformance. Open architectures facilitate systematic governance around a national vision of expectations of how digital applications and services function and act together across a digital health enterprise, which facilitates coordinated planning and investment, as well as modular upgrading of digital solutions.

To empower better care, a FHIR-based architecture requires high-quality health content to flow through it. To this end, a steadily growing portfolio of OPEN CONTENT—such as clinical guidelines, health worker training courses, decision support logic, minimum data dictionaries and computable care plans—is available for adaptation and deployment. One example is the WHO SMART Guidelines [28], an approach to documenting requirements and encoding practice guidelines as computable content using FHIR implementation guides (Health Level Seven International 2021b), CQL and ICD, which can then be readily loaded into compliant applications [50]. SMART guidelines have been published or are being developed on topics such as antenatal care, family planning, HIV, immunization and pediatric emergency care. Additional normative agencies are beginning to adopt this approach of publishing open content: the Centers for Disease Control and Prevention (CDC) has launched their ‘Adapting Clinical Guidelines for the Digital Age’ initiative [4]; and the National Commission for Quality Assurance (NCQA) has encoded digital quality metrics (dQMs) using FHIR and CQL [32]. Having a shared, open library of high-quality resources advances the dissemination and use of evidence-based recommendations to drive person-centered care across solutions. Open content encoded to such standards forms a universal representation of health, data and calculations, facilitating interoperability and consistent implementation regardless of software. This mitigates against vendors, products and platforms locking-in content to their specific digital solutions; and additionally makes possible conformance testing, verifying that solutions perform according to specifications and standards [19, 53].

Executing on this next-generation full-STAC vision—the concurrent implementation of open standards, technologies, architecture and content—will require multi-stakeholder collaboration across the public and private sectors, including policymakers, funders, advisors, technologists and professional and government agencies. To enable this collaboration, digital health governance is a key foundation to coordinate stakeholders and policies that enable a well-functioning health system. Digital health governance defines who, what and how to govern to achieve health service impact [25]. Patients also need to be involved to build systems that respond to their needs and preferences and that they will trust. Strong leadership at the national, regional and global levels, coupled with enforced procurement policies requiring specific interoperability standards, local workforce development and capacity building, will be essential for turning this vision into a reality on the ground. Investing today in an interoperability standards-based open-architecture ecosystem will pay long-term dividends for person-focused health systems, even in the most resource-constrained environments [54]. Importantly, this open approach means there is room and opportunity for all digital health stakeholders at the table. By aligning with the full-STAC approach, existing digital platforms can also advance next-generation data-driven solutions and help accelerate country-led digital transformation.

We foresee a future where individuals' health data travels with them; where local developers, technologists and organizations are better equipped to build next-generation solutions for local problems; where health providers are assisted by timely evidence-based digital content; where high-quality longitudinal datasets power representative and appropriate artificial intelligence models that advance care; and where ministries of health generate governed real-time insights into their populations' health. This is the culmination of the vision that 194 WHO Member States endorsed in the Global Strategy on Digital Health.

Countries are at different stages of maturity in their digital health journey, including optimizing of systems and interventions; a full-STAC approach unlocks potential throughout. Notably, this does not require that countries start from scratch. Benefits can also be achieved through augmentation of ongoing digital investments. Governments hold an essential role in reinforcing the full-STAC approach: guiding and enforcing interoperability standards-based procurement; establishing governance standards for health data; specifying interoperability and open-access standards to which all digital health services and applications conform; and stipulating regulatory standards for medical devices and medical products that use digital technologies. Countries spearheading this movement will be critical to the success of its widespread use and resulting value across the health sector. The full-STAC approach is a remedy to ongoing entropy in digital health, providing critical ingredients to enhance benefits from digital transformation of health systems for the successful implementation of the Global Strategy on Digital Health. Critically, it will place countries in the driver’s seat in devising strategic use of digital technologies to address populations’ most pressing healthcare challenges.

## CONFLICT OF INTEREST

The authors declare that they have no conflicts of interest regarding the research, authorship and/or publication of this article. Authors are participating in their personal capacity and the views expressed in this publication are those of the authors and do not necessarily represent the views of the funding agencies, the companies, or institutions mentioned.

## AUTHOR’S CONTRIBUTIONS

G.M., M.S., F.H. and C.L. wrote the original draft and contributed to the conceptualization, review and editing of the manuscript. M.B., K.K., M.L., J.P.N., M.S., D.S., N.R. contributed to the conceptualization, review and editing of the manuscript. S.B., R.D., A.M., Y.M., J.N., V.N., M.O., D.R.O.G., D.R.O., S.R., J.T., B.T., S.W., A.L., M.G. contributed to the review and editing of the manuscript. P.L. contributed to the conceptualization of the manuscript.

## DATA AVAILABILITY

No new data were generated or analyzed in support of this research.

## Footnotes

Health systems that are interoperable can: “(i) reduce health care costs associated with redundant diagnostic testing, unnecessary hospitalizations and preventable readmissions; (ii) make better use of resources and management to know how, when and where those resources are used; (iii) effectively monitor notifiable diseases, seasonal diseases, communities’ disease burden and other aspects; (iv) aid public health research; and (v) strengthen disaster response.” [3]
