# Correction to: A full-STAC remedy for global digital health transformation: open standards, technologies, architectures and content

**DOI:** 10.1093/oodh/oqae048

**Published:** 2024-12-19

This is a correction to: Garrett L Mehl, Martin G Seneviratne, Matt L Berg, Suhel Bidani, Rebecca L Distler, Marelize Gorgens, Karin E Kallander, Alain B Labrique, Mark S Landry, Carl Leitner, Peter B Lubell-Doughtie, Alvin B Marcelo, Yossi Matias, Jennifer Nelson, Von Nguyen, Jean Philbert Nsengimana, Maeghan Orton, Daniel R Otzoy Garcia, Daniel R Oyaole, Natschja Ratanaprayul, Susann Roth, Merrick P Schaefer, Dykki Settle, Jing Tang, Barakissa Tien-Wahser, Steven Wanyee, Fred Hersch, A full-STAC remedy for global digital health transformation: open standards, technologies, architectures and content, *Oxford Open Digital Health*, Volume 1, 2023, oqad018, https://doi.org/10.1093/oodh/oqad018

The author Alvin B Marcelo was incorrectly listed as Alvin D Marcelo. This has been corrected online.

